# Evaluation of in Vivo Response of Three Biphasic Scaffolds for Osteochondral Tissue Regeneration in a Sheep Model

**DOI:** 10.3390/vetsci6040090

**Published:** 2019-11-09

**Authors:** Alberto M. Crovace, Alessia Di Giancamillo, Francesca Gervaso, Laura Mangiavini, Davide Zani, Francesca Scalera, Barbara Palazzo, Daniela Izzo, Marco Agnoletto, Marco Domenicucci, Corrado Sosio, Alessandro Sannino, Mauro Di Giancamillo, Giuseppe M. Peretti

**Affiliations:** 1 Department of Emergency and Organ Transplantation, University of Bari Aldo Moro, 70010 Bari, Italy; 2 Department of Veterinary Medicine, University of Milan, 20122 Milano, Italy; alessia.digiancamillo@unimi.it (A.D.G.); davide.zani@unimi.it (D.Z.); mauro.digiancamillo@unimi.it (M.D.G.); 3 Department of Engineering for Innovation, University of Salento, 73100 Lecce, Italy; francesca.gervaso@unisalento.it (F.G.); francesca.scalera@unisalento.it (F.S.); barbara.palazzo@unisalento.it (B.P.); danielaizzo84@gmail.com (D.I.); alessandro.sannino@unisalento.it (A.S.); 4 CNR NANOTEC, Institute of Nanotechnology c/o Campus Ecotekne, via Monteroni, 73100 Lecce, Italy; 5 IRCCS Istituto Ortopedico Galeazzi, Via R. Galeazzi 4, Milan, 20122 Milano, Italy; laura.mangiavini@unimi.it (L.M.); marcoagno@hotmail.it (M.A.); sosiocor@gmail.com (C.S.); giuseppe.peretti@unimi.it (G.M.P.); 6 Department of Biomedical Sciences for Health, University of Milan, 20122 Milan, Italy; 7 Residency Program in Orthopaedics and Traumatology, University of Milan, 20122 Milan, Italy; marcodomen@gmail.com

**Keywords:** osteochondral defects, scaffold, biomaterials

## Abstract

Osteochondral defects are a common problem in both human medicine and veterinary practice although with important limits concerning the cartilaginous tissue regeneration. Interest in the subchondral bone has grown, as it is now considered a key element in the osteochondral defect healing. The aim of this work was to generate and to evaluate the architecture of three cell-free scaffolds made of collagen, magnesium/hydroxyapatite and collagen hydroxyapatite/wollastonite to be implanted in a sheep animal model. Scaffolds were designed in a bilayer configuration and a novel “Honey” configuration, where columns of hydroxyapatite were inserted within the collagen matrix. The use of different types of scaffolds allowed us to identify the best scaffold in terms of integration and tissue regeneration. The animals included were divided into four groups: three were treated using different types of scaffold while one was left untreated and represented the control group. Evaluations were made at 3 months through CT analysis. The novel “Honey” configuration of the scaffold with hydroxyapatite seems to allow for a better reparative process, although we are still far from obtaining a complete restoration of the defect at this time point of follow-up.

## 1. Introduction

Articular cartilage and subchondral bone form a well-integrated system, with unique biomechanical properties, that provides the efficient transmission of significant static and dynamic loads [1,2]. This osteochondral tissue may frequently undergo damage by trauma and disease; as a consequence, the joint undergoes an osteoarthritic degeneration leading to severe pain, joint deformity, and loss of joint motion [3,4,5,6,7]. The degeneration may initiate at different locations. It could start from the subchondral bone, which becomes weaker and unable to support a proper loading, causing a consequent cartilage degeneration [8,9,10]. On the other hand, the lesion may begin on the cartilage tissue, therefore inducing altered stimulus to the subchondral bone, which becomes sclerotic [9]. Both scenarios may be responsible for modifying the performance of the osteochondral structure. The osteochondral tissue has a poor spontaneous regenerative potential, due to the scarce presence of multipotent cells in cartilage and due to the insufficient migration of cells from the bone marrow; thus, when damaged, it repairs with a tissue characterized by low biomechanical and biological properties. Its treatment represents a major challenge in the human and veterinary orthopedic field and has driven different lines of research, both in vitro and in orthotopic models [11,12,13,14,15,16]. In the clinical practice, several solutions have been proposed for osteochondral repair: the most common techniques are based on the recruitment of multipotent cells from the bone marrow [11,17]. However, the repairing tissue is mainly fibro-cartilaginous and does not exhibit the mechanical properties of native hyaline cartilage [17]. Osteochondral transplantation has been widely used as autologous tissue and as allograft [18,19,20,21,22,23]. However, in recent years regenerative medicine is taking advantages not only of traditional implants, but also of engineered biocompatible parts, including degradable porous scaffolds in some cases integrated with cells or molecules. Indeed, several scaffolds have been developed for osteochondral lesions [23] combining cell-free strategies and the use of different layers to mimic the bone-cartilage structure [12,24,25,26,27]. It is worth noting that bone and osteochondral scaffold design must enable hierarchical composites porous structures to attain desired mechanical function and mass transport, and to produce these structures within arbitrary and complex three-dimensional (3D) anatomical shake [27]. Our paper attempts to give a contribute in this field, proposing new osteochondral scaffold configurations and assessing their in vivo behavior. 

Particularly, the aim of this study was to evaluate, at three months follow up, the regenerative performances of three cell-free scaffolds in collagen and Mg doped hydroxyapatite or in collagen and hydroxyapatite/wollastonite in a sheep model. In our study, two different three-dimensional collagen/HA osteochondral substitutes were proposed. In the first configuration, called “Bi-layer”, a highly porous ceramic scaffold was surrounded in circumference by a thin layer of collagen, and characterized by a stable pre-integration between the osteo (ceramic) and chondral (collagen) components, easily surgically handled, and compliant at the implant site. In the second configuration, called “Honey”, highly porous columns of hydroxyapatite were inserted within the cylindrical collagen scaffold. The preparation of these different scaffolds is presented in this paper and the preliminary results following their implantation in a sheep model were also reported.

## 2. Materials and Methods 

### 2.1. Scaffold Design and Realization 

The inorganic component, both for the Bi-layer and for the Honey configurations, was synthesized with the foam replication method [28,29,30,31,32,33,34,35] and composed of Mg doped hydroxyapatite (Mg-HA, HMG group) or wollastonite/hydroxyapatite (WS/HA, HWS group) mixtures. Mg-HA was prepared replacing a 10% molar of calcium ions, keeping the (Ca + Mg)/P = 1.67. WS/HA scaffold was prepared by sintering WS/HA mixtures powders in a 1/1 ratio. The Bi-layer scaffolds were then produced accordingly to the procedure previously reported [36] and the bioceramic pillars were embedded in the collagen type I layer [37,38] to form the biphasic Honey scaffolds [39]. The organic component, made of collagen, was fabricated by freeze-drying a collagen solution (2% wt/v) following a previously reported protocol [32,33]. All biphasic substitutes were sterilized in oven under vacuum at 160 °C for 2–4 h before implantation. All configurations tested, with corresponding sample names and materials used to produce the scaffold (both cartilage and bone substitute) are reported in Table 1.

Bi-layer and Honey scaffolds have been characterized in terms of (i) ceramic phase composition by X Ray Diffraction (XRD); (ii) morphology by scanning electron microscopy (SEM Zeiss Evo40, Jena, Germany) and micro CT analysis; and (iii) mechanical resistance by uniaxial compression test. 

The XRD pattern was registered with a D-Max/Ultima diffractometer (Rigaku, Tokyo, Japan) using CuKa radiation (λ = 1.5418A°) in the step scanning mode recorded in the 2θ range of 20–60°, with a step size of 0.02° and step duration of 0.5 s. 

All the tomographic acquisitions were performed by a GE Phoenix Nanotom CT system equipped with a 180 kV/15 W nanofocus X-ray tube and a 2300 × 2300 pixel on 12 bit Hamamatsu flat panel detector. A molybdenum target, suitable for weak absorbing specimens, was used for all the analyzed specimens. The experimental conditions for the acquisition are summarized in Table 2. The volume reconstruction was carried out with the proprietary application software Phoenix datos|x 2 reconstruction. The 3D visualization and analysis software Avizo 8 Fire Edition of Visualization Sciences Group (a company belonging to the FEI group) were used for the image processing of the datasets.

The compression tests were performed using a mechanical analyzer (Lloyd LR5K instrument, AMETEK Test & Calibration Instruments, Bognor Regis, UK) and five samples, both Bi-layer and Honey, compressed at a crosshead speed of 0.5 mm/min and the maximum stress calculated. For each experimental group, means were assessed by descriptive statistics. Each experimental group was compared with the control group (CTRL) and standard Student’s *t*-test was performed. Statistical significance was defined as a *p*-value of <0.05.

After the approval of the Italian Ministry of Health and in strict accordance with the recommendations in the Guide for the Care and Use of Laboratory Animals (D.L. 26/2014) of the National Institutes of Health, the sheep selected for the experimental treatment were identified and stabled for 30 days for acclimatization. 

### 2.2. Management of the Animals and Surgical Procedures

**Figure 1:**
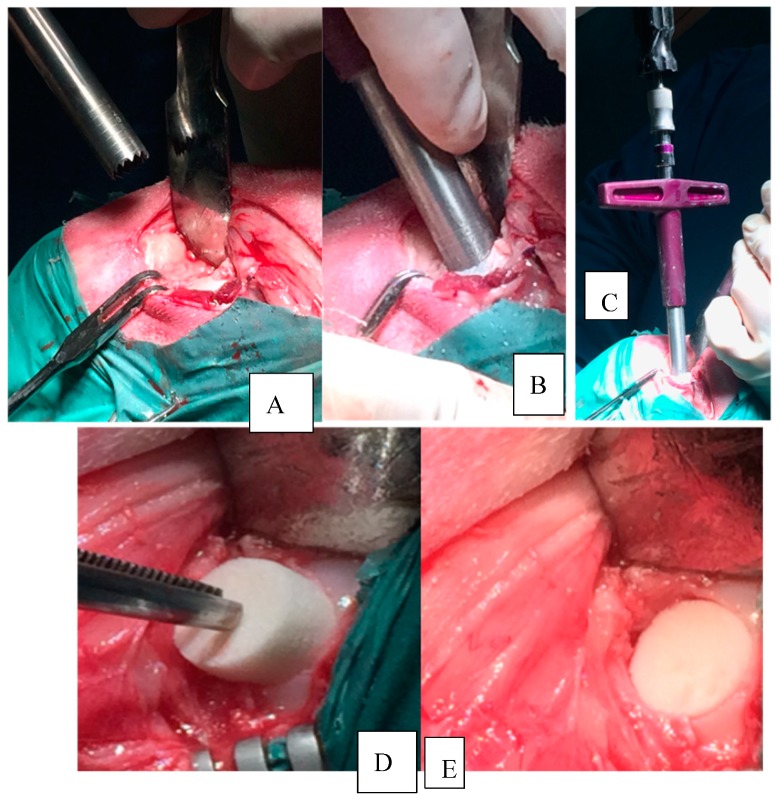
Osteochondral lesion practiced with custom made instruments. (**A**) Core millimeter punch before its application. (**B**) Core millimeter punch inserted to a depth of 9 mm. (**C**) Hand drill inserted in the sleeve. (**D**) Scaffold inserted with press-fit technique. (**E**) Scaffold implanted in situ.

All surgeries were performed by the same team under aseptic conditions and both general and spinal anesthesia was made to minimize pain and suffering. Eighteen sheep, Bergamasca breed, female 50 ± 4 Kg, 6 years old, were recruited and fasted for 24 h, then an intravenous catheter was placed in the jugular vein. The sheep were sedated with ± midazolam (MIDAZOLAM 5 mg/mL IBI, Aprilia, Italy, 0.4 mg/kg IV) and they were also treated with flunixin meglumine (FLUNIXIN 50 mg/mL, Norbrook, Agri Laboratories, UK, 1 mg/kg IV). When the desired sedation was reached, the animals were placed on the right lateral decubitus to receive spinal anesthesia. The lombo-sacral area was surgically scrubbed and the spinal anesthesia was performed through a subarachnoideal injection using lidocaine (LIDOCAINA 2%, Esteve Veterinaria SPA, Italy, 2 mg/kg) and buprenorphine (BUPRENODALE 0.3 mg/mL, Dechra, Italy, 300 mcg); during the procedure the sheep received oxygen through a nasal catheter and eventually also a bolus of propofol (PROPOFOL 1%, Fresenius Kabi, Germany, 1 mg/kg IV) was administered. During the surgery, sheep received fluids to a mean speed of 10 mg/kg/h adapting it to the condition of the patient. Then, sheep were placed in dorsal recumbence with the right knee in flexion. The skin in the area was trimmed and scrubbed. After the disposition of surgical drapes, a longitudinal incision of about 5–7 cm was made on the medial aspect of the right knee, in the projection zone of the medial condyles. A capsulotomy and a partial removal of the Hoffa body were performed to obtain a better exposition of the condyle. Subsequently, with a hyperflexion of the knee to better expose the right medial condyle, an osteochondral lesion (11 mm of diameter and 9 mm in depth) was generated on the load bearing part of the condyle, measuring with custom made instruments (Core Punch, Sleeve, Hand drill). The lesion was neat and surrounded by healthy cartilage. The scaffolds were inserted by press fit (Figure 1).

The joint capsule and the subcutaneous tissue were closed with absorbable sutures and the skin was closed with non-absorbable sutures. A waterproof medication was placed on the lesions; the animals were free to move without any bandage or immobilization tool. Postoperative X-ray were practiced in all treated animals to evaluate the position of the scaffolds. All operated animals received Benzylpenicillin procaine (IZOTRICILLINA 100 mL, IZO SRL, Italy, 10 mL of a 200,000 UI/mL solution IV) and flunixin (FLUNIXIN 50 mg/mL, Norbrook, Agri Laboratories, UK, 1 mg/kg IV) every 24 h for seven days.

The animals after the surgery were stabled in the Section of Veterinary Clinics and Animal Production of the Department of Emergencies and Organ Transplantation (D.E.O.T.) of the University of Bari. Every day during the experimental phase the animals were clinically controlled. 

### 2.3. Protocol of the Study

The defects in three sheep were left untreated (CTRL), while the remaining 15 sheep were divided into 3 groups: 5 lesions were treated with a biphasic scaffold made of collagen type I for the cartilaginous phase and small cylinders of magnesium hydroxyapatite embedded in collagen type I for the osseous component (HMG); 5 lesions were treated with a biphasic substitute formed by collagen type I and wollastonite (BWS); 5 lesions were treated with a scaffold made of collagen type I and small cylinders of wollastonite/hydroxyapatite embedded in collagen type I (HWS). We performed the power calculation for a two-tailed t test with power of 0.95, an alpha error of 0.05 and an effect size of 0.86, using freely available software (G*Power Version 3.0.10; University of Düsseldorf, Germany) [40]. The results of this analysis suggested that a minimum of six sheep per group would be sufficient to detect significant differences between groups.

All animals were euthanized after 3 months from surgery by 50 mL of a potassium chloride solution administered IV after general anesthesia was induced with propofol (PROPOFOL 1%, Fresenius Kabi, Germany) IV. Death of the animal was confirmed by the absence of heart beats by ECG monitoring and absence of thoracic excursions.

For all animals, the treated leg was harvested without opening the knee joint, placed in a portable refrigerator box and sent to Veterinary Medicine University of Milan in Lodi to undergo CT evaluation. Subsequently, the tissues underwent gross analysis and histological examination [39].

### 2.4. CT Evaluation

The CT scans were performed on the harvested knees at the Department of Veterinary Medicine (DIMEVET), Ospedale Didattico Universitario Az. Polo Veterinario di Lodi. In particular, for the CT scans a GE BrightSpeed 16 slice CT machine was used. The acquisition protocol was performed as follows: helical mode, slice thickness 1.25 mm, 120 kVp, 200 mA, pitch 0.93, convolution kernel “bone plus”. 

### 2.5. Macroscopic Evaluation

The skin and the articular capsule were opened, and macroscopic assessment of the knees was performed by using the International Cartilage Repair Society (ICRS) Macroscopic Score, which evaluates these specific parameters: leveling, stiffness, integration, degree of repair and overall macroscopic aspect of the defects. Three different observers independently scored the samples [39]. 

## 3. Results

### 3.1. Pre-Implant Osteochondral Scaffolds Characterization

**Figure 2:**
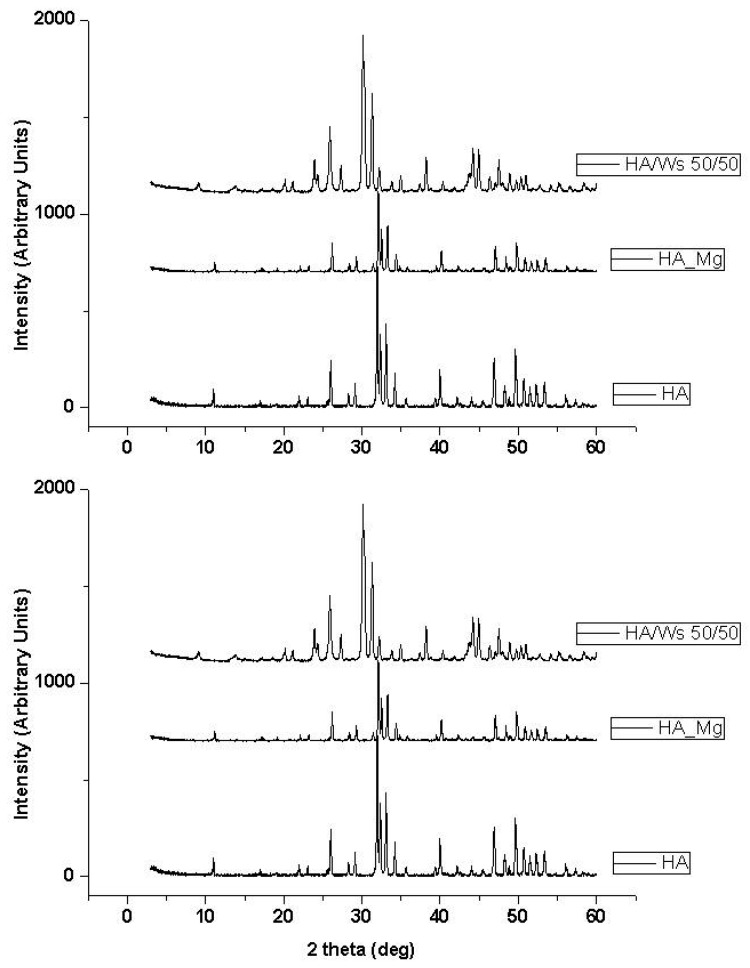
X-ray diffraction pattern of pure hydroxyapatite (HA), magnesium-doped hydroxyapatite (HA_Mg), hydroxyapatite/wollastonite composite (HA/WS) ceramic scaffold. X-ray diffraction pattern of pure hydroxyapatite (HA) ceramic scaffold has been reported for comparison.

Two ceramic powders were synthesized to produce the “bone” scaffolds of the whole substitutes implanted in the present study: namely, magnesium-doped hydroxyapatite (MG) and hydroxyapatite/wollastonite composite (WS). In order to check if the desired material composition was obtained, an X-ray diffraction analysis (XRD) was performed. The diffraction pattern of the synthesized ceramic powders confirmed the obtainment of the established MG and WS that are stable at high sintering temperature (Figure 2). More in detail, MG underwent a slight dissociation in secondary phases (mainly calcium oxide and magnesium phosphates), during the sintering processes, while WS scaffolds could be fabricated and sintered without significant decomposition or phase changes with respect to the starting materials.

**Figure 3:**
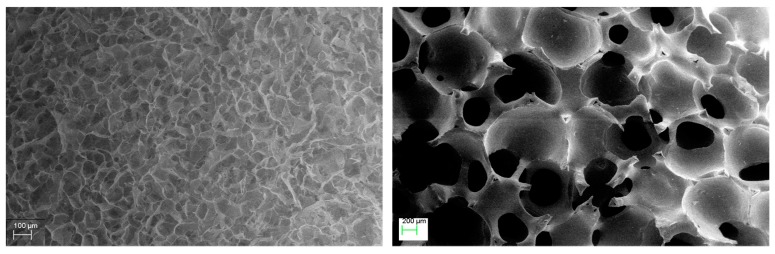
SEM images of the collagen component (**left**) and of ceramic component (**right**) of Bi-layer configuration and Honey configuration.

Both collagen (organic, made by freeze-drying) and ceramic (inorganic, made by sponge replica) scaffolds were observed by SEM to check the ceramic and collagen scaffold pore size and porosity interconnection. Figure 3 shows the SEM images of the collagen (left) and ceramic (right) scaffold where the highly interconnected porosity of both materials is highlighted. The collagen scaffold shows an average pore size of about 100 μm, suitable to host chondrocytes, while the ceramic scaffold has higher pore size of about 500 μm of diameter that should allow the new bone formation within the ceramic structures [37,38,41].

**Figure 4:**
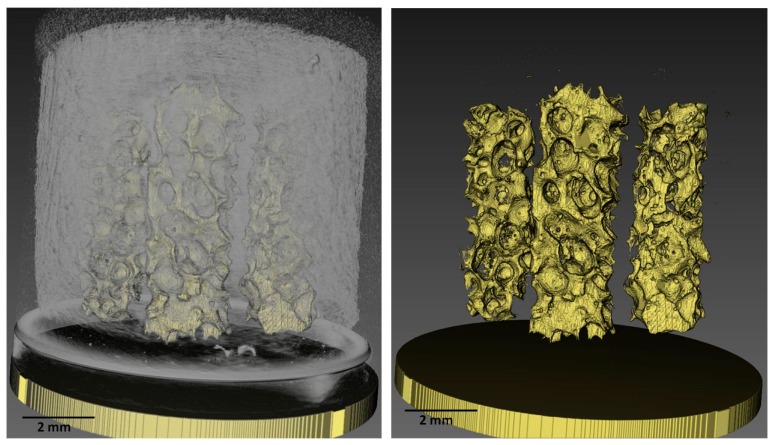
MicroCT images of the whole Honey scaffold (**left**) and of the ceramic pillars (**right**). Scale bar = 2 mm. (Please note that images are not orthogonal projections but are in perspective and as a consequence of it the scale bar can give only indicative information).

The microCT analysis performed on the Honey configuration (Figure 4) allowed checking the correct positioning of the ceramic pillars and the whole porous structure of the substitute.

**Figure 5:**
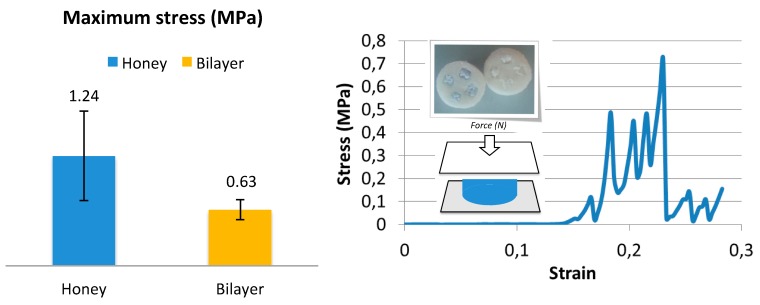
Maximum stress at failure of both Honey and Bi-layer configurations tested in compression (**left**) and a typical stress–strain curve of a Honey scaffold (**right**) in which it is possible to distinguish four main peaks corresponding to the four pillars break.

The initial mechanical resistance of an osteochondral substitute is crucial. However, the substitutes proposed here are not designed to bear the whole physiological load, rather to offer the proper environment for cell colonization and proliferation. On the other hand, scaffolds must sustain surgical handlings without any breaks. Mechanical tests in compression were hence performed to check the stress at break of the two configurations, Bi-layer and Honey, primarily to verify the mechanical resistance of the small ceramic pillars and compare them to the Bi-layer resistance. The average values of maximum stress of Honey and Bi-layer scaffolds (n = 5) are reported in the histogram of Figure 5 (left). Honey scaffolds showed a higher average value of stress at failure than Bi-layer; however, the difference was not significant, due to the high standard deviation. The stress–strain curve of both Honey and Bi-layer scaffold configurations has the typical behavior of fragile materials, showing a rapid and linear increase of stress as function of strain followed by a sudden break. Interestingly, in the stress–strain curve of Honey scaffold, we distinguished four main peaks corresponding to the break of the four small pillars embedded within the collagen matrix (Figure 5, right).

### 3.2. In Vivo Results

All surgical procedures were performed without any major complication.

All animals treated were able to stand on their four legs and to bear the load on the operated limb 2–4 h after the surgery.

### 3.3. Radiological Evaluation

**Figure 6:**
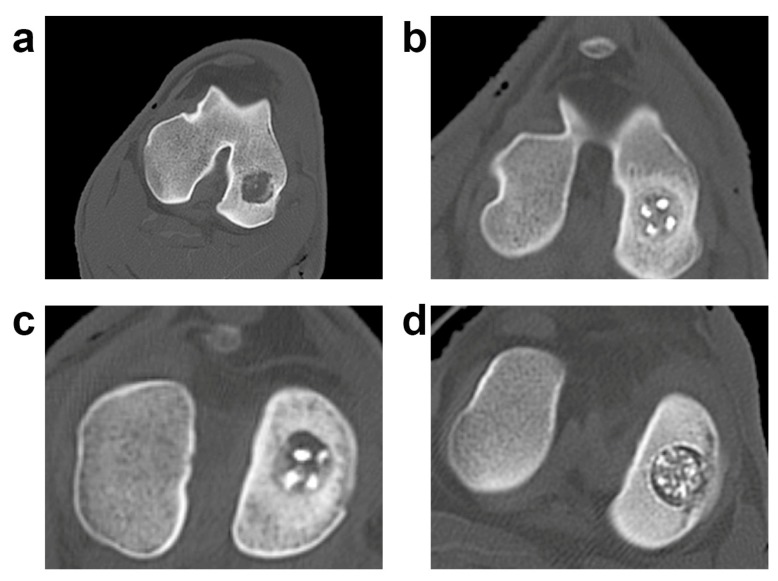
CT evaluation of the three biphasic scaffolds. (**a**) Radiologic pictures of the experimental groups; a representative lesion for each experimental group is shown. Control (CTRL): (**a**); HMG: (**b**); HWS: (**c**); BWS: (**d**).

CT scans showed the persistence of the bony scaffold, well integrated with the surrounding bone as shown in our previous publication [39] (Figure 6).

### 3.4. Macroscopic Evaluation

**Figure 7:**
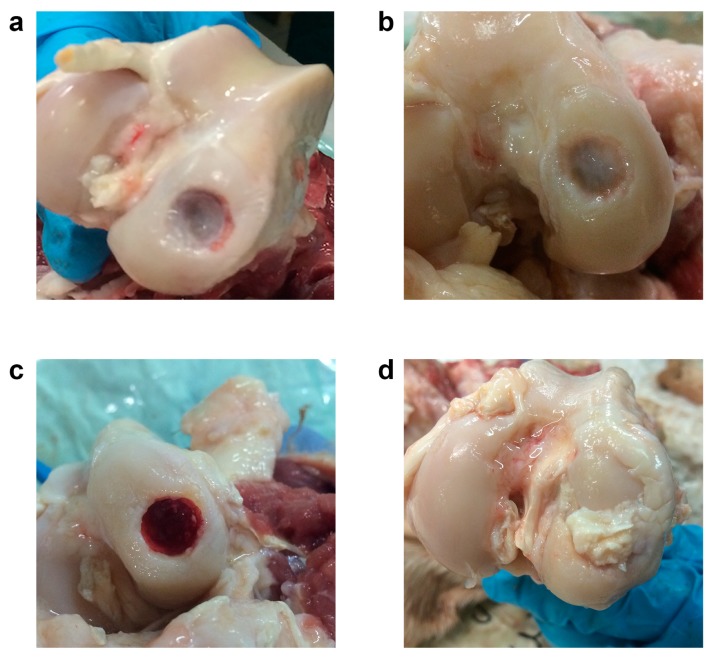
Macroscopic evaluation of the three biphasic scaffolds. A representative lesion for each experimental group is shown. CTRL: (**a**); HMG: (**b**); HWS: (**c**); BWS: (**d**). The filling level of the experimental samples was different, and it was characterized by a poor amount of reparative tissue.

After performing the radiological evaluation, the sheep joints were dissected and macroscopically evaluated with ICRS evaluation as previously shown [39]. The CTRL group was characterized by the almost complete absence of reparative tissue, whereas the HMG lesion was partially filled with tissue. Finally, the presence of wollastonite in the other two groups seemed to interfere negatively with the reparative process (Figure 7).

## 4. Discussion

In this study, three kinds of cell-free biphasic scaffolds were analyzed as potential novel materials for repairing osteochondral lesions. In previous studies from our group, an osteochondral substitute seeded with stem cells [42] or a biphasic scaffold seeded with chondrocyte and a cell-free scaffold were compared in a swine orthotopic model [30,36]. Surprisingly, the superiority of the unseeded scaffold was evidenced in the short-term follow-up. Cell-free samples showed the presence of fewer cells, but characterized by a chondrocyte-like phenotype. The osteochondral lesion repair in this model, though, was far from complete. It was therefore decided to change the biphasic scaffold’s architecture in order to improve the reparative potential and the integration of the substitute with the healthy tissue. Three biphasic scaffolds with different designs and compositions were compared in this project: the aim was to improve the scaffolds’ architecture to favor integration of the scaffold with healthy tissue and to promote the acquisition of a chondrocyte phenotype of migrated cells. In particular, two different scaffold configurations were designed and morphologically and mechanically characterized before implantation. The SEM analysis performed on collagen and ceramic scaffolds showed that porosities of both substitutes are highly interconnected and present an average pore size suitable for cartilage and bone regeneration, respectively [29,37,38]. The mechanical properties in compression of the novel configuration with four HA pillars embedded in the collagen matrix resulted comparable or even higher of those of the Bi-layer configuration with a unique HA cylinder. 

All biphasic scaffolds with different designs were successfully implanted on a sheep model without any major adverse event. In this study, none of the groups showed a complete and successful repair of the osteochondral lesion. However, the HMG configuration gave the best results in terms of integration and repair potential. The presence of wollastonite in the other two scaffolds may have interfered with the healing process of the osteochondral lesion. 

Moreover, our data successfully demonstrated the biocompatibility of all substitutes. 

However, the experimental time (3 months) is not sufficient for a complete repair of an osteochondral lesion. Thus, we are planning to analyze longer time points (6 months and 1 year). Additionally, considering the shrinkage of some experimental samples, we also believe that these data clearly suggested the need of coupling these materials with active molecules which may stimulate the repair and regeneration of cartilaginous and bony tissues.

## 5. Conclusions

Highly interconnected three-dimensional collagen/hydroxyapatite scaffolds were manufactured by replica method using a polyurethane sponge. They showed mechanical properties superior to those reported in literature, demonstrating the efficacy of their sintering. Collagen scaffolds were successfully fabricated by a freeze-drying technique. The construct porosity could be properly regulated by varying the design parameters. Cross-linking treatments can improve the mechanical properties of the collagen scaffold. The integration of the hydroxyapatite and the collagen of the osteochondral substitute was properly obtained. The sheep model in vivo study, designed to assess the effectiveness of the proposed scaffolds for repairing osteochondral defects, demonstrated the biocompatibility of three biphasic cell-free scaffolds in an osteochondral defect. The new “Honey” configuration of the scaffold with hydroxyapatite enabled better reparative process, although a complete restoration of the defect is still far from being achieved. A new study, designed with the employment of the HMG scaffold in smaller lesions, could represent a future attempt for correcting the incomplete repair. In order to stimulate the migration of endogenous reparative cells and favor the reparative process, the addition of active molecules or growth factors will also be considered, thus demonstrating the potentials of this new biphasic cell-free substitute for the repair of osteochondral defects.

## Figures and Tables

**Table 1:** Design of osteochondral scaffold used in the in vivo study. For each configuration tested, sample names and the materials used to produce the scaffold (both cartilage and bone substitute) are reported.

Osteochondral Scaffold Sketch		Osteochondral Scaffold Configuration	Sample Name	Scaffold Material	
Top View	Side View			Cartilage Scaffold	Bone Scaffold
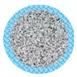	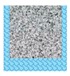	Bilayer	BWS	Collagen	Blend WS/HA
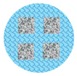	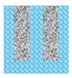	Honey	HWS	Collagen	Blend WS/HA
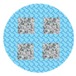	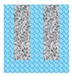	Honey	HMG	Collagen	Mg-doped HA
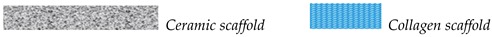					

**Table 2:** Experimental conditions for the tomographic acquisition.

Voltage (kV)	Current (µA)	Voxel Size (µm^3^)	Exposure Time (s)	Projections	Scan Time (min)
65	145	12.5 × 12.5 × 12.5	1.250	1400	176
